# Electronic cigarette social norms among adolescents in New South Wales, Australia

**DOI:** 10.1093/heapro/daae018

**Published:** 2024-03-03

**Authors:** Amelia Yazidjoglou, Christina Watts, Grace Joshy, Emily Banks, Becky Freeman

**Affiliations:** Centre of Epidemiology for Policy and Practice, National Centre for Epidemiology and Population Health, Australian National University, Mills Road, Acton, Canberra, Australian Capital Territory, 2601, Australia; The Daffodil Centre, The University of Sydney, A Joint Venture with Cancer Council NSW, Dowling Street, Woolloomooloo, Sydney, New South Wales, 2011, Australia; Centre of Epidemiology for Policy and Practice, National Centre for Epidemiology and Population Health, Australian National University, Mills Road, Acton, Canberra, Australian Capital Territory, 2601, Australia; Centre of Epidemiology for Policy and Practice, National Centre for Epidemiology and Population Health, Australian National University, Mills Road, Acton, Canberra, Australian Capital Territory, 2601, Australia; Prevention Research Collaboration, Sydney School of Public Health, Faculty of Medicine and Health, The University of Sydney, John Hopkins Drive, Camperdown, Sydney, New South Wales, 2050, Australia

**Keywords:** electronic cigarettes, social norms, descriptive norms, injunctive norms, e-cigarette marketing, social exposure

## Abstract

The use of electronic cigarettes (e-cigarettes) is common and increasing, especially among youth. In 2022/2023, 30% of 12- to 17-year-olds reported ever using e-cigarettes in Australia—a >50% increase from 2017 (14%). Several adverse e-cigarette health effects have been identified and most effects remain unknown. Social norms, rules that govern social behaviours, are associated with current and future adolescent e-cigarette use. Understanding social norms in Australian adolescents is critical to the development of targeted and effective e-cigarette prevention activities. This study aims to explore e-cigarette social norms among adolescents living in New South Wales, Australia. A total of 32 online single or paired semi-structured qualitative interviews were conducted involving 46 participants aged 14–17 years, as part of the Generation Vape project. Reflexive thematic analysis was applied within a constructivist perceptive. Adolescents perceived e-cigarettes use as prolific among their peers, with use considered common, acceptable and normal. Fuelled by social exposure to e-cigarettes, ‘everyone’ was generally thought to be using them (descriptive norms). E-cigarette use was considered so entrenched that it was part of adolescent identity, with abstinence regarded as atypical. Use was driven by an internalised desire to fit it (injunctive norm), rather than being attributed to overt/external ‘peer-pressure’. Positive e-cigarette norms exist among Australian adolescents with norm formation strongly influenced by social exposure, including e-cigarette promotion. Prevention efforts should include limiting adolescent exposure to e-cigarette marketing to help redefine existing pro-e-cigarette social norms and protect health.

**Contribution to Health Promotion**

- Social norms are associated with e-cigarette initiation and future use, and although adolescents are particularly susceptible to the health impacts of e-cigarettes and the use of e-cigarettes is increasing, little evidence on adolescent social norms exist.
- Adolescents perceived e-cigarette use as common and normal—abstinence was atypical—which often served as social proof validating their e-cigarette use.
- Use was driven by an internal desire to fit in rather than external peer-pressure.
- Norms were strongly influenced by social exposure, including e-cigarette promotion and marketing.
- Prevention efforts must reshape adolescent social norms and therefore behaviour by restricting exposure to e-cigarette advertising.

## BACKGROUND

In Australia, 30% of 12- to 17-year-olds reported ever having used electronic cigarettes (e-cigarettes) in 2022/2023, a >50% increase from 2017 (14%) (Scully *et al*., 2023). A sample of 14- to 17-year-olds living in New South Wales, Australia’s most populous state, found that 32% had ever used e-cigarettes in 2021 (Watts *et al*., 2022). These high prevalences of use are in spite of laws preventing the sale or supply of e-cigarettes under 18-year-olds.

Electronic nicotine delivery systems, also known as electronic cigarettes, vapes or e-cigarettes (used henceforth), are a diverse group of battery-powered or rechargeable products that generate a heated aerosol for users to inhale (National Academies of Sciences Engineering and Medicine, 2018). Originally manufactured in China in 2003 and introduced into the United States and European markets in 2006/2007, e-cigarettes have been quickly adopted by tens of millions of users across the globe (Laverty *et al*., 2018; Jerzyński & Stimson, 2023). Since their inception, e-cigarettes have rapidly evolved with millions of products now available with most markets dominated by large volume, high nicotine concentration disposable devices (Zhu *et al*., 2014; Williams and Talbot, 2019; SCHEER, 2021; US Food and Drug Administration, 2022; Diaz *et al.*, 2023.

While many important health effects of e-cigarettes remain unknown, several health risks have been identified including: addiction; poisonings both intentional and accidental; nicotine toxicity from inhalation; burns and injuries; lung injury and increased smoking uptake in non-smokers (Baenziger *et al*., 2021; Banks *et al*., 2023). Adolescents are particularly vulnerable to the impacts of nicotine. During this critical developmental period, adolescents undergo rapid and extensive biological changes such that any disruption may result in both short- and long-term health consequences (US Department of Health and Human Services, 2016). Evidence from adolescent smokers suggests nicotine can elicit changes in neural reward systems that increase the risk of comorbid substance abuse and reward-seeking behaviours, and decrease aversion behaviours (US Department of Health and Human Services, 2016). Adolescent e-cigarette use has been found to be associated with depression, mood disorders, attention-deficit/hyperactivity disorder and conduct disorders, however, the relationship between e-cigarettes and mental health remains unclear and is likely to be complex and multidirectional (Becker *et al*., 2021; Nguyen and Mital, 2022). Given the health impacts of e-cigarettes, is it vital to understand the drivers behind adolescent e-cigarette use.

Social norms are central to the formation of attitudes and behaviours, are known to influence smoking initiation and cessation and are common intervention points for traditional tobacco control policy (Mead *et al*., 2014; Chung and Rimal, 2016; East *et al*., 2021). They are the rules recognised by members of a group that determine what is normal and expected to guide or restrict social behaviour (Chung and Rimal, 2016). The two most common social norms studied in relation to smoking and e-cigarettes are descriptive—the perceived prevalence and patterns of use—and injunctive—perceived pressure to conform to avoid social sanction or gain social approval (Mead *et al*., 2014; Chung and Rimal, 2016; Camenga *et al*., 2018). Descriptive and injunctive norms are bound in a cyclical relationship whereby they both inform, guide and reinforce each other and the social practice. Social norms are not innate, universal or static but the embodiment of information from the individual’s social network, and the physical and symbolic environment known collectively as social exposure (Mead *et al*., 2014).

There is increasing evidence that indicates positive e-cigarette norms (including social acceptability and high perceived prevalence) are associated with intention to use, initiation and continued use of e-cigarettes among youth (Mantey *et al*., 2016; Camenga *et al*., 2018; Amin *et al*., 2020; Thoonen and Jongenelis, 2023). Favourable norms concerning the perceived ‘coolness’ of both devices and e-cigarette users themselves have been suggested to enhance users’ social desirability and popularity which may influence adolescent behaviour (Ranjit *et al*., 2021; Smith *et al*., 2021). Qualitative data on adolescent norms are very limited with a 2021 meta-ethnography identifying 13 studies—conducted in the USA, the UK and Canada—finding four emerging norms: understanding of addiction, harm perception and parental and peer perceptions (Smith *et al*., 2021). Norms can be rapidly generated, entrenched and broken in response to external events and pressures (Cislaghi and Heise, 2018) and in order to effectively leverage e-cigarette social norms to reduce social acceptability and use of e-cigarettes, it is vital to first understand what norms are present, most persistent and influential among adolescents.

To our knowledge, no studies in Australia have explored social norms concerning e-cigarettes among adolescents (14–17 years). This study aims to gain insight into the complex and nuanced social norms articulated by adolescents to improve understanding of the influence of norms on e-cigarette behaviour. Given the adverse health effects of nicotine and the widespread youth use of e-cigarettes, understanding the juncture between norms and use will be fundamental for effective and targeted tobacco control activities for the protection and improvement of adolescent health.

## METHODS

This study uses data from the Generation Vape research project, a 3-year (2021–24) study examining e-cigarette use among young Australians. This article reports on the second wave of qualitative data collection (March–April 2022), involving online single or paired semi-structured interviews with 14- to 17-year-olds residing in New South Wales. The interviews were designed to explore awareness, perceptions, attitudes and knowledge among young people. Ethics approval for the project was granted by the University of Sydney Human Research Ethics Committee (Project number: 2021/442) in July 2021.

All respondents with a self-reported age of 14–17 years (inclusive) living in New South Wales who had heard of e-cigarettes were eligible for study selection irrespective of e-cigarette use status (no other eligibility criteria). Recruitment was conducted by a professional research recruitment agency via online panels with equal quotas of ever e-cigarette users and never-users, males and females and private and government schools and 75% metropolitan participants, to reflect the general population geographical distribution. Recruitment bias may be present, as only those subscribed to the online panels and willing to be interviewed were included, however, this is unlikely to significantly impact the results as generalisability of the result is not necessary and the study is exploratory in nature. Both parental and participant consent were obtained after they had received a participant information statement.

A total of 32 interviews including 46 participants—25 males and 21 ever-users—were conducted. Interviews were ~30 minutes in length and conducted via Zoom by two interviewers using a piloted discussion guide. Interviews were audio recorded, transcribed verbatim and de-identified. The reflexive thematic analysis process, as outlined in Braun and Clarke, was selected for data synthesis as it focuses on the development of patterns of meaning (themes) while acknowledging the active role of the researcher in analysis (Braun and Clarke, 2022). The reflexive thematic analysis process is non-linear as it is not necessary to follow the phases in order or complete each phase before progressing. Rather, the process is flexible and recursive by allowing the return to previous phases and enabling the completion of several phases concurrently (Braun and Clarke, 2022).

The analytical process involved several phases (Braun and Clarke, 2022). The first phase, familiarisation of the dataset, was implemented by repeated listening of audio recordings and reading of transcripts with general notes recorded. Coding, the second phase, was conducted manually with codes, associated text excerpts and participant characteristics copied into an Excel spreadsheet. Coding was inductive—driven by the key patterns identified in the dataset and without the use of a pre-specified coding structure, due to the exploratory nature of the research and the desire to capture all experiences relating to social norms. Codes were generally semantic—relating to the explicit meaning of the data—with latent or higher-level interpretations recorded in an accompanying note. Multiple codes were applied to an excerpt where applicable. Coding for half the dataset was completed by one author then revised and discussed by all authors for consistency, appropriateness and completeness. The remaining transcripts were coded with the revised set of codes and the first half was recoded to collect data captured by any new codes that arose through author discussions. Upon the completion of coding, initial themes were generated by a single author (phase three of the reflexive thematic analysis process). Coded extracts were grouped under candidate themes by clustering those with similar or related meanings separated by norm type. Several codes were collapsed, some divided and others reworded to construct sub-themes and themes.

Phases four and five, developing and reviewing themes and refining, defining and naming themes, occurred concurrently with the input from all authors. During this process, some sub-themes were identified as sufficiently prominent to be considered themes while other sub-themes were abandoned to improve overall clarity. The final phase, writing, included structuring the themes and integrating extracts into a logical and meaningful narrative guided by the research aims.

This phenomenological study was influenced by a constructivist ontology and interpretivist epistemology reflecting the philosophical paradigm that there are multiple subjective and socially constructed realities governed by people’s social interactions and that knowledge is subjective and individual (Al-Saadi, 2014; Bannister-Tyrrell and Meiqari, 2020; Brown and Dueñas, 2020). It also recognises that the researcher is not independent of the analysis but a situated interpreter in which the researcher’s own assumptions and perspective will influence the findings (Al-Saadi, 2014).

## RESULTS

### Descriptive norms

Descriptive norms refer to the perceived prevalence and patterns of use. For this analysis, common behaviour is considered to refer to the perceived frequency of e-cigarette use and normalcy the degree to which the behaviour is expected, usual or typical.

#### Common behaviour

All participants considered e-cigarette use to be ‘very common…like a lot of people do vape’ with many commenting that ‘everyone’s tried a vape’ and that ‘it’s everywhere’ and ‘so prominent’ among their age group. One participant suggested that it was so common that out of his acquaintances, he couldn’t name one that had not used an e-cigarette:

*Um, people who don’t, don’t do it, I guess, to be honest I haven’t met many people who haven’t done it.* (E-cigarette user, male, 14–15 years)

Observing overt and frequent e-cigarette use by their peers was sometimes described as a justification for use. One participant found that the visibility of e-cigarettes was so influential that she expressed her disbelief at the notion that individuals could choose not to partake:

*I was just like well everyone around me does. Like did you not expect me to do it once?* (E-cigarette user, female, 14–15 years)

For other participants, they were able to rationalise or validate their e-cigarette use by thinking that the widespread use negated many of the negative aspects of e-cigarettes:

*You see everyone doing it and like you know, you see like oh, you know what, it’s not too bad*. (E-cigarette user, female, 16–17 years)

Whilst it was generally considered that most peers were using e-cigarettes, some recognised that there were substantial numbers of peers that didn’t use e-cigarettes:

*Yeah, there’s a lot of people that don’t do it at all …Yeah, it just depends. So it just depends on like the people… there’s not… there’s like… it’s about 70% of people… ah 80% - no even more… like 90% of people in my friend group just don’t vape…* (E-cigarette user, male, 14–15 years)

#### Normalcy

The perception that e-cigarette use was common was often linked to the normalisation of the behaviour and the positioning of use as standard:

*… it’s so common now, it’s kind of like, normalised…Like, it’s like, standard to do it. It’s not like taboo or anything like that. So, people, everyone just does it.* (Non-e-cigarette user, female, 16–17 years)

E-cigarette use was not just tolerated by participants as being the norm but was often condoned and accepted:

*Like, we’re not gonna tell off somebody [for vaping].* (E-cigarette user, female 16–17 years)

*It keeps becoming more standard like, normalised and accepted.* (Non-e-cigarette user, female, 16–17 years)

In fact, for some participants, not using e-cigarettes was considered unusual behaviour with use inextricably linked to a normal teenager’s identity.

*if you’re not [vaping]… it’s kind of like a little bit like you’re not like a normal teenager.* (E-cigarette user, female, 16–17 years)

### Injunctive norms

The injunctive norms relate to how one ought to act to gain social approval while avoiding social sanction. For the purpose of this analysis, peer-pressure is conceptualised as overt and external pressure while a desire to fit in captures inferred, subtle and internally constructed pressures to conform.

#### Desire to fit in

Almost all participants associated e-cigarette use with a desire to fit in. Though this desire did not necessarily apply to themselves, non-users felt others’ use was motivated by this need to fit in while some e-cigarette users personally experienced using e-cigarettes to fit in. E-cigarette use was viewed as a defining feature of some groups that acted as a unifying behaviour fostering a shared identity and sense of belonging.

*I guess a kind of sense of like belonging, cause if like a lot of people there are doing it… like to fit-in.* (Non-e-cigarette user, female, 16–17 years)

*I guess it kinda is, something that brings people together in a way. Like it brings them together you know? They’re all sharing one you know, and they’re talking.* (E-cigarette user, male, 14–15 years)

This was deeply embedded in some groups such that it was considered part of the group’s culture.

*But like it’s kinda, they do it cos they’re trying to, it’s part of their, culture, in a way.* (E-cigarette user, male, 14–15 years)

This desire to fit in was described as an internal pressure compelling people to use e-cigarettes:

*They put the pressure on themselves to be like ‘oh if I don’t do this… they’re gonna not be friends with me’.* (Non-e-cigarette user, male, 16–17 years)

Another element of fitting in demonstrated by participants was the desire to avoid social sanction such that there was a reluctance to confront their peers using e-cigarettes or to report them to authority figures:

*like even if someone in the stall next to me was doing it in the bathroom, it’s not like I’m gonna be like ‘oh my god, I’m gonna go tell the principal’, like I’d just walk out*. (Occasional e-cigarette user, female, 16–17 years)

#### Peer-pressure

There were a few accounts of participants experiencing or observing peer-pressure to use e-cigarettes:

*Probably peer-pressure, I guess… I just didn’t wanna say no in that situation*. (Former regular user, male, 16–17 years)

*I think someone just… tells them to do it… like they get pressured into it. Cause I’ve seen someone got pressured into it*. (Non-e-cigarette user, male, 16–17 years)

However, some rejected the idea that people were using e-cigarettes due to peer-pressure and suggested other motivations such as curiosity as a driving force behind use:

*Um, it wasn’t peer-pressure or anything, it was just, curiosity*. (Occasional e-cigarette user, female, 14–15 years)

Similarly, others that refuted peer-pressure as a driver instead suggested it was the accessibility, availability and perceived prominence of e-cigarettes that led to their e-cigarette use:

*I don’t know if it was like peer-pressure, I think it was just mainly because like I hadn’t before… and I knew that like a lot of people did like it…* (Former occasional user, female, 16–17 years)

*not not that I’m someone who you know just easily gets peer-pressured into things, um not in any way… um but it was just something where… it was a… like a situation where it was easy… to give it a go.* (E-cigarette user, male, 16–17 years)

One participant who questioned the influence of peer-pressure went as far as suggesting that her decision to try e-cigarettes was based on the necessity, and even inevitability, of using e-cigarettes.

*Like it wasn’t peer-pressure or anything. It was just one of my friends had them… I just thought I wanna experience it cos one day I’m gonna have to anyway.* (Tried e-cigarettes, female, 14–15 years)

Furthermore, a person’s individual choice (either to use or abstain) was generally accepted and respected by participants with many considering e-cigarette use by others none of their concern:

*you do you, I’ll do me.* (Ex-e-cigarette user, female, 16–17 years)

*I feel like most people kind of just mind their business… but…there would be some people who would… probably like question it, but wouldn’t necessarily like speak up and go like snitch or whatever.* (Non-e-cigarette user, female, 16–17 years)

## DISCUSSION

This study describes the current descriptive and injunctive social norms pertaining to e-cigarette use among adolescents in New South Wales. It details the diversity of adolescent experience highlighting seemingly contradictory views illustrating the depth and complexity of social norms. Peer e-cigarette use was considered prolific, commonplace and standard to the extent that abstaining was atypical. E-cigarette use among this age group was regarded as so entrenched that it had become a fixture of their adolescent identity and a shared, and at times defining, characteristic of their cohort. An acute internal desire to ‘fit in’ seems to be driving e-cigarette use. Peer-pressure, while recognised, was deemed less influential on motivation to use e-cigarettes.

The prevalence of peer e-cigarette use is exaggerated in adolescents’ perceptions with many considering either most or even everyone to be using e-cigarettes. In addition to the data used for this analysis, Generation Vape also conducted a concurrent quantitative survey within the same population and found that 32% of 14- to 17-year-olds living in New South Wales reported ever e-cigarettes use—while this is still a high degree of use, it is far lower than adolescents’ perception. This divergence is present elsewhere with ~61% of US middle and high school students overestimating peer e-cigarette prevalence (Agaku *et al*., 2019). The portrayal of prolific and widespread e-cigarette use erroneously posits that a high prevalence alternate reality exists from which descriptive norms are then founded (Mead *et al*., 2014; Liu *et al*., 2020). The discord between actual and perceived prevalence highlights that the descriptive norm is susceptible to—and in this case, greatly influenced by—other sources of social information, such as e-cigarette promotion and advertising, rather than actual prevalence (Agaku *et al*., 2019).

In their study investigating the discordance between perceived and actual e-cigarette prevalence among grade 6–12 students in the USA, Agaku *et al*. found that as exposure to e-cigarette advertising increased, so did the proportion of adolescents overestimating e-cigarette prevalence (Agaku *et al*., 2019). Of those adolescents with the most e-cigarette advertising exposure, 78% overestimated prevalence compared to only 47% among those with the least advertising exposure. They also reported that those who overestimated e-cigarette prevalence had higher odds of being curious about e-cigarettes (odds ratio 3.29; 95% CI 2.41–4.48) than those who did not overestimate prevalence (Agaku *et al*., 2019). Adolescents may overestimate prevalence as repeated, frequent and wide exposure to positive e-cigarette marketing gives the impression the use is more common, popular and acceptable than in reality (Liu *et al*., 2020; Zheng and Lin, 2021). News articles that contain seemingly neutral text, such as the reporting of statements regarding the large diversity in devices and flavours, can inadvertently frame products as popular or desirable reinforcing the idea that e-cigarettes are common and widespread (Duong and Liu, 2019). In addition to media exposure, exposure to e-cigarette use in public can also contribute to overestimations with those exposed to e-cigarette use in public more likely to overestimate use than those not exposed (adjusted odds ratio 1.83; 95%CI 1.29–2.58) due to the implicit acceptability conveyed by such public and overt use (Agaku *et al*., 2020). To curb adolescent e-cigarette use, it is imperative that this overestimation be challenged through population health policies that limit adolescent exposure to e-cigarette use in public settings and in marketing and promotion, through policies that reduce the supply of e-cigarettes to adolescents and through education.

Descriptive norms can also serve as a decisional short-cut guiding behaviour when the situation is ambiguous (i.e. perceiving e-cigarettes as widespread and common sets this as the default behaviour), or as ‘social proof’ of the social acceptance of a behaviour (Chung and Rimal, 2016). Social proof is an example of a mechanism in which descriptive norms can inform and reinforce injunctive norms and refers to the concept that widespread prevalence and popularity of a given behaviour translate to a broader acceptability of the behaviour, and therefore, must be the correct way to behave (Mead *et al*., 2014; Chung and Rimal, 2016). There is growing evidence of e-cigarette acceptability among adolescents and in England, Canada and the USA, ~40%, 46% and 47% of 16- to 19-year-olds believe that their friends approve of e-cigarette use (East *et al*., 2019). Our study demonstrates the presence and pervasiveness of ‘social proof’ as many justified their own e-cigarette use on the basis that ‘everyone else’ was using e-cigarettes and some found the social proof so compelling that using e-cigarettes was inevitable.

Both peer-pressure and a desire to fit in embody the injunctive norm (how one ought to behave). Peer-pressure, conceptualised as overt and external pressure to conform in a particular manner by one’s peers, is often referenced in parental and educational materials as a challenge confronting adolescents, with many of these resources including approaches that aim to empower adolescents to decline e-cigarettes in the face of external peer-pressure (Lung Foundation Australia, 2021; Healthdirect Australia, 2023). Although peer-pressure was noted by some in our study, an internal and individually constructed desire to ‘fit in’ was most salient. Thus, while peer-pressure may be a motivator, it was not identified by most participants as a key driver for e-cigarette use. Fairman *et al*. report that in their study of US adolescents and their parents peer-pressure was identified by parents but not adolescents in relation to e-cigarette use (Fairman *et al*., 2021). Attributing adolescent e-cigarette use to peer-pressure does not seem to reflect adolescents’ predominant experiences in both our study and Fairman and colleagues thus challenging this explanation for use (Fairman *et al*., 2021). Overemphasis on overt peer-pressure from adolescents on others to use e-cigarettes places the burden of responsibility on adolescents while downplaying the persistent and insidious influence of industry marketing and promotional activities, including the design of the products themselves.

The inclusion of e-cigarettes as a characteristic of adolescent identity has been created, promoted and perpetuated by e-cigarette advertising. E-cigarettes social media posts, primarily produced by vape shops, e-cigarette company representatives or advocates—including paid influencers—convey the products as edgy and performative while encouraging individuality through diverse device and flavour choices, and allude to the ability of e-cigarettes to enhance social capital and acceptance (McCausland *et al*., 2019; Struik *et al*., 2020). These depictions provide the social information used by adolescents to construct their impression of a typical teenager and help to guide their behaviour to gain social approval while avoiding social sanction.

‘Cool’ is often referenced in relation to e-cigarettes as a perceived benefit (positive expected outcome) or reason for use (Romijnders *et al*., 2018), in e-cigarette promotion and advertising (Lee *et al*., 2023), general media discourse (Bellafante, 2018; Hoffman, 2018) and even health messaging (Sydney Children’s Hospitals Network, 2022). Cool is difficult to define. The meaning of cool—what is or is not cool, and who personifies cool—is ambiguous, subjective, group-specific and ever-changing. Increasingly e-cigarettes are considered ‘uncool’ by adolescents (Smith *et al*., 2021)—a sentiment that resonated with participants in our study. In 2022, only 2.4% of a sample of never-smokers aged 11–17 years in Great Britain reported cool as the reason for use, this is compared to 65.4% that reported it was just to give it a try (curiosity) (Action on Smoking and Health, 2022). Acceptance of the idea that adolescents use e-cigarettes because they are cool or that it makes adolescents appear cool fails to address specific actionable points of intervention for policymakers. It masks and minimises the importance of definable and distinct influences on adolescent e-cigarette use such as product characteristics, marketing and promotion and use of e-cigarettes in public spaces all of which inform e-cigarette social norms.

Current norm research has focused primarily on the influence of interpersonal relationships on norm perceptions while evidence of the impact of physical and symbolic environments on norm development is lacking (Duong and Liu, 2019). The physical environment—capturing the physical attributes or cues in a setting or physical attributes of the products themselves—can also readily inform descriptive and injunctive norms. Smoking in public spaces among other situational factors is known to influence smoking norms and behaviour (Mead *et al*., 2014) and, results from this study indicate e-cigarette norms can be similarly influenced as evidenced by the normalisation and justification of adolescent e-cigarette use, including on the basis of widespread and commonplace public use. To help protect adolescent health and prevent the initiation and regular use of e-cigarettes, current favourable adolescent descriptive norms must be challenged and reshaped by policies limiting exposure in public spaces which promote alternate norms whereby use is perceived to be uncommon and atypical.

In relation to the symbolic environment, media including online advertising, point of sale promotion, product placement in movies and other entertainment, while assumed to be important for norm formation, remains chronically under-researched particularly in relation to e-cigarettes (Mead *et al*., 2014; Duong and Liu, 2019) despite four in five youths reporting exposure to e-cigarette marketing in the USA in 2016 (Marynak *et al*., 2018). The tobacco and e-cigarette industries employ aggressive and relentless marketing strategies, some of which are specifically designed to target youth. In the USA in 2022–23, Juul Labs conceded over $1 billion to settle lawsuits made by 45 states and $1.7 billion to local governments and consumers that claimed the company marketed their high nicotine e-cigarettes to minors (Australian Broadcasting Corporation, 2022; The Guardian, 2023). The investigation found that Juul recruited thousands of social media influencers and used young-looking models to deliberately target children and teens (Australian Broadcasting Corporation, 2022). The results of this study provide further insight into the detrimental influence of industry marketing and promotion. This is most clearly evidenced by the adulteration of the adolescent identity where some participants consider it is now a requirement to use e-cigarettes to be considered typical and to fit in. Addressing and limiting the degree of social exposure, particularly exposure from physical and symbolic environments, to e-cigarettes will be paramount in efforts to redirect adolescent social norms and behaviours.

Understanding what social norms exist and how they interact with one another, and the social norms specific to adolescents is crucial for the development of effective tobacco and e-cigarette control initiatives. E-cigarette use driven by an internal desire to fit in and compounded by erroneously overestimated prevalence and pervasive social proof, has established e-cigarette use as the norm for Australian adolescents. Understanding these norms, their ability to inform one another and the drivers of these norms is fundamental to the development of effective and targeted tobacco control activities. The evidence outlined here suggests that messaging that focuses on addressing peer-pressure or framing e-cigarettes as uncool may not resonate appropriately, as they do not reflect the lived experiences of our youth. Instead, dispelling ideas of widespread use, challenging social proof and preventing exposure to e-cigarette marketing and promotion is more likely to impact youth use. The Australian *National Tobacco Strategy 2023–2030* has recently been released and outlines several objectives, two of which are particularly pertinent to e-cigarette social norms among adolescents: to prevent the uptake of e-cigarettes by young people and non-smokers and limit the marketing and use of e-cigarettes (Department of Health and Aged Care, 2023). As such, the *Strategy* recognises the responsibility of Federal, and State and Territory governments to prohibit e-cigarette advertising, promotion and sponsorship (Department of Health and Aged Care, 2023). These actions have the ability to reduce exposure, reshape social norms and protect health and it will be important to monitor and evaluate the success of these efforts.

This study sampled only adolescents living in New South Wales and, as social norms are group-specific such that certain norms in one group may not persist in another, it is important not to uncritically apply these findings to other populations or contexts. As with any qualitative study, online recruitment may have resulted in some community members not being sampled. Social norms by nature are changeable and dynamic and therefore, these findings reflect the norms at a given period of time and are likely to evolve as social practices, regulation, health promotion and data on health effects change. Ongoing research will be needed to ensure norms remain current in the face of a changing policy landscape, the rapid development of products and industry innovations in the promotion and marketing of products alongside other social and political changes. Furthermore, future research is needed to explore social norms in other adolescent populations, including Aboriginal and Torres Strait Islander populations and culturally and linguistically diverse communities. A better understanding of the functioning of e-cigarette injunctive and descriptive norms will assist in the creation and effective targeting of health promotion policy and interventions to address youth e-cigarette use.

## CONCLUSIONS

This study, an Australian first, describes the descriptive and injunctive social norms relating to e-cigarettes among adolescents. Prolific peer e-cigarette use is perceived, with use considered commonplace and normal. E-cigarettes have become so instrumental to adolescents’ psyche and identity that abstinence is considered atypical. A desire to fit in was a key motivator for use. Social exposure involving favourable e-cigarette promotion and use in public settings is likely to have strongly influenced the formation of these norms. Given the association of current and future e-cigarette use with positive norms and the health consequences of e-cigarettes, reshaping adolescent social norms and therefore behaviour by restricting exposure to e-cigarette advertising will be vital to e-cigarette prevention efforts and the improvement of adolescent health.

## Data Availability

Data from interviews are unavailable for sharing due to ethical requirements.
